# Disruption of the integrin-linked kinase (ILK) pseudokinase domain affects kidney development in mice

**DOI:** 10.1016/j.jbc.2021.100361

**Published:** 2021-02-02

**Authors:** Nada Bulus, Kyle L. Brown, Glenda Mernaugh, Anika Böttcher, Xinyu Dong, Charles R. Sanders, Ambra Pozzi, Reinhard Fässler, Roy Zent

**Affiliations:** 1 Department of Medicine, Division of Nephrology, Vanderbilt University Medical Center, Nashville, Tennessee, USA; 2 Center for Matrix Biology, Vanderbilt University Medical Center, Nashville, Tennessee, USA; 3 Center for Structural Biology, Vanderbilt University, Nashville, Tennessee, USA; 4 Department of Molecular Medicine, Max Planck Institute of Biochemistry, Martinsried, Germany; 5 Institute of Diabetes and Regeneration Research, HelmholtzZentrum, Munich, Germany; 6 Department of Biochemistry, Vanderbilt University, Nashville, Tennessee, USA; 7 Veterans Affairs Hospital, Nashville, Tennessee, USA

**Keywords:** molecular dynamics, cell culture, cell adhesion, western blot, molecular modeling, CD, collecting duct, DMEM, Dulbecco's minimal essential media, ILK, integrin-linked kinase, IPP, ILK–pinch–parvin, MD, molecular dynamics, PBS, paxillin-binding site, PC, principal component, PH, pleckstrin homology

Integrin-linked kinase (ILK), a central component of the intracellular ILK–pinch–parvin complex, localizes together with paxillin to focal adhesions and regulates integrin-mediated cell functions. ILK was initially misclassified as a kinase based on phenotypical characterization of cells expressing ILK mutated in the “kinase” domain, such as the E359K and K220M mutants and a V386G/T387G mutation in the paxillin-binding site (PBS). ILK is now known to be a pseudokinase, and mechanisms of action of these mutants are not clear. We selectively induced expression of only the E359K, PBS, and K220M ILK mutations in the developing kidney collecting system and kidney collecting duct (CD) cells and analyzed their impact on structural integrity using molecular dynamics (MD) simulations. Mice or CD cells carrying the E359K mutation had a severe phenotype that is indistinguishable from ILK-null mice or ILK-null CD cells. The K220M mutant mice developed normally, and K220M-CD cells had a mild adhesion, migration, and tubulogenesis defect. The PBS mutant mice had a subtle developmental defect, and PBS-CD cells had moderate functional abnormalities. Consistent with these observed phenotypes, MD studies suggest that the E359K mutant produces the most structurally perturbed, and K220M the most WT-like ILK molecules. Although all three mutations disrupted ILK binding to parvin and paxillin *in vitro*, only the E359K mutation decreased ILK binding to pinch suggesting that it increases ILK misfolding. Thus, point mutations in the ILK pseudokinase domain cause functional abnormalities by altering the ILK structure, leading to increased turnover and destabilization of ILK–parvin and (sometimes) ILK–pinch interactions.

The integrin-linked kinase (ILK)–pinch–parvin (IPP) complex is a critical component of focal adhesions that binds to the cytoplasmic tail of the integrin β subunits. Integrins, composed of an α and a β subunit, are the principal receptors that mediate cell–extracellular matrix interactions and regulate many cell functions, including adhesion, spreading, migration, polarization, and tubulogenesis. ILK is a 450 amino acid multidomain pseudokinase protein consisting of an N-terminal domain with five ankyrin repeats, a COOH-terminal pseudo kinase domain, and an intervening pleckstrin homology (PH) domain (1, 2, 3, 4). Pinch interacts with the ankyrin repeat domain, while parvin family members bind to the pseudokinase domain. The three components of the IPP complex are thought to assemble in the cytosol, and the pseudokinase domain is required for this interaction to occur. The pseudokinase domain is inherently unstable, and the heat shock protein Hsp90 is required to stabilize interactions between parvin and ILK (5). The expression of all three IPP components is critical for IPP complex formation as genetic deletion of either pinch or ILK leads to a decrease in IPP complex formation; however, the mechanism for this interdependency is unknown (2).

ILK is critical for survival in mice and its constitutive loss results in lethality at the periimplantation stage due to abnormal epiblast polarity and adhesion (6). Variable phenotypes occur with tissue-specific deletion of ILK, which causes branching morphogenesis defects in the developing kidney collecting system (7) and mammary gland (8). A point mutagenesis strategy was employed to define the mechanisms of ILK function in the setting of the IPP complex. Mice carrying a constititve and homozygous R211A or the S343A/D mutations in the pseudokinase domain are normal, while mice with a K220A/M mutation in the same domain show kidney developmental abnormalities due to decreased interactions between ILK and α-parvin caused by increased destabilization of the ILK pseudokinase domain structure (9). ILK has also been proposed to bind directly to the cytosolic signaling protein paxillin *via* a highly conserved paxillin-binding site (PBS) found in the pseudokinase domain of ILK as well as several other proteins (10, 11, 12). Mice carrying the V386G/T387G mutation in the PBS-binding sites die at E9.5, and *in vitro* studies revealed that these mutations alter ILK stability and decrease its ability to bind α-parvin or paxillin. Finally, an E359K mutation was made based on a Glu-Arg salt bridge that connects coevolved motifs that defines the highly conserved fold found in in all eukarytoic protein kinases (13). However, it is now clear that ILK is not a kinase and the mechanism of action of this mutant deviates from structurally homologus eukaryotic protein kinases, thus its effects *in vivo* must now be regarded as unknown (11, 14, 15, 16, 17).

We previously showed that deleting ILK in the developing ureteric bud (UB) of the kidney results in a branching morphogenesis defect and that ILK-null collecting duct (CD) cells have abnormalities in multiple integrin-dependent functions (7). In this study, we generated mutations in the developing UB and CD cells to define the mechanisms whereby K220M, E359K, and PBS mutations alter ILK function in the setting of polarized epithelium. We subsequently employed molecular dynamic (MD) simulations and molecular modeling techniques to visualize how these ILK mutations can disrupt the IPP complex.

## Results

### The E359K, PBS, and K220M ILK mutants differentially affect UB development

ILK is required for normal development, and global ILK-null mice die at the periimplantation stage (6). Mice constitutively expressing K220M die shortly after birth from kidney development complications (9), and PBS ILK mutants do not survive longer than E9.5 (12). We generated the constitutive E359K mutant mice as described in the Experimental procedures and Figure S1 and found that they have an early embryonic lethal phenotype (data not shown).

To compare the differential effects of the E359K, K220M, and PBS mutations in the same biological system *in vivo*, we expressed the mutant ILK versions in the UB, which gives rise to the kidney collecting system. This was achieved by intercrossing the heterozygous E359K, K220M, and PBS mutant mice with ILK-floxed mice expressing Cre recombinase under the hoxB7 promoter (18). The heterozygous mutant mice and heterozygous floxed ILK mice crossed with hoxB7 Cre mice were normal (data not shown). The breeding strategy used should result in 12.5% of the offspring expressing the mutant protein in the UB. Littermates carrying the floxed and mutated ILK alleles without the Cre recombinase transgene served as controls for experiments. The PBS and K220M mutant mice were born in the correct Mendelian ratio while the E359K mutants were born at a lower ratio (6.7%), which was similar to the mice lacking ILK in the UB (8.1%), suggesting that some of these mice died *in utero* or at birth. The PBS and K220M mice developed normally and did not display overt developmental phenotypes. By contrast, the surviving E359K and the UB-ILK null mice died at about 8 weeks of age from renal failure.

**Figure 1:**
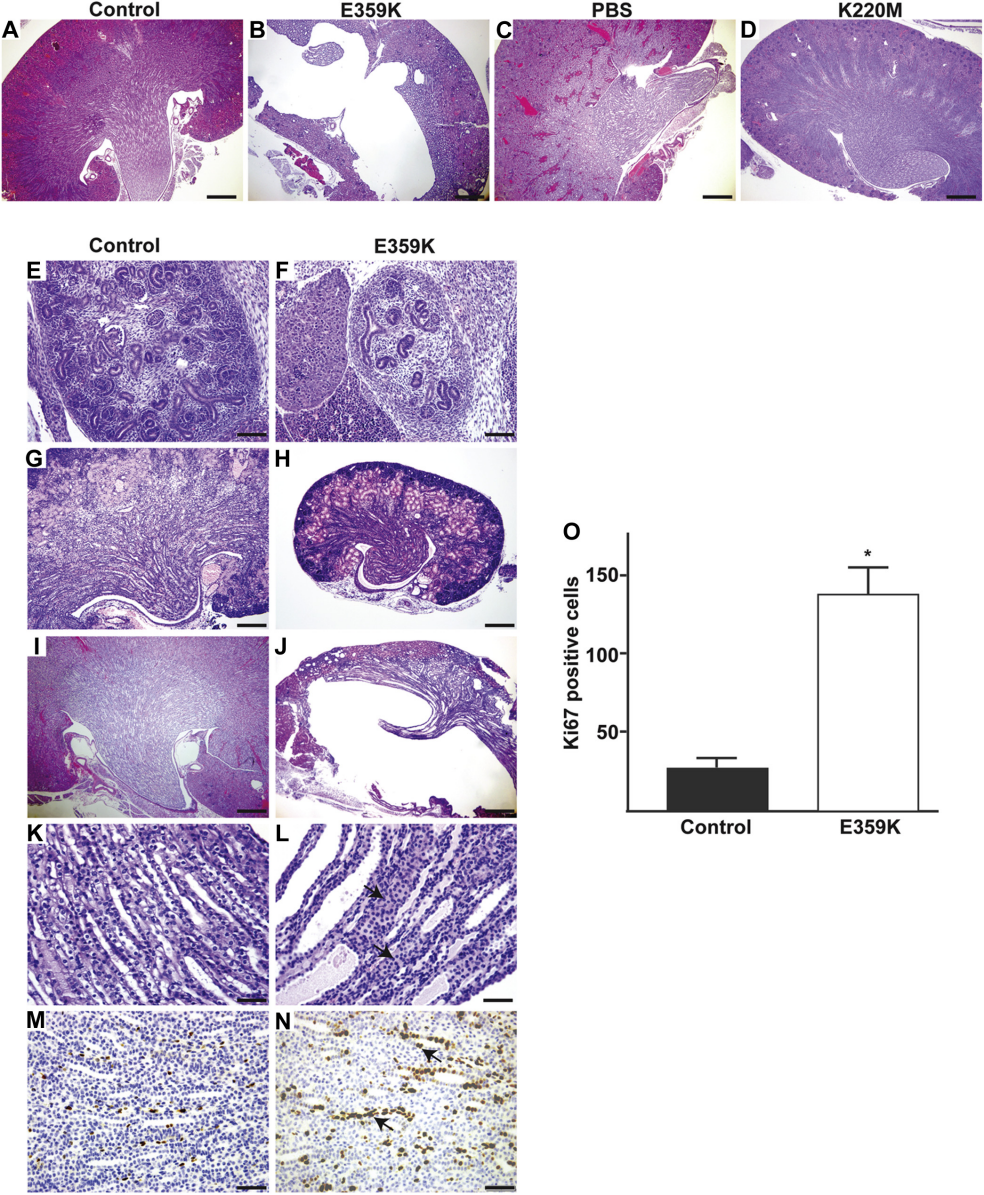
**ILK mutations in the developing collecting system of murine kidneys.***A–D*, images of kidneys from control and E359K, PBS, and K220M mutant mice generated as described in the Experimental procedures. *A*, *C*, and *D* are kidneys from 1-year-old mice, *B* is from a 6-week-old mouse (Scale bar is 200 μm). *E–L*, abnormalities in the E359K kidneys were compared with controls at E15.5 (*E* and *F*), P1 (*G* and *H*) and at 4 weeks of age (*I* and *L*). A moderate branching morphogenesis defect and impaired metanephric mesenchyme induction are present at E15.5 (*E* and *F*). Newborn kidneys were hypoplastic and dysplastic (*G* and *H*), and they became dilated by 4 weeks of age due to intraluminal obstruction of the collecting ducts by collecting duct cells (*arrow*) (*I*–*L*) (Scale bar 100 μm in *E*, *F*; 200 μm in *G*–*J* and 50 μm in *K*–*L*). *M* and *N*, during the postnatal period, the tubules displayed intratubular proliferation characterized by increased Ki67 cells (*arrow*) (Scale bar 50 μm). *O*, the number of Ki67 cells per high powered field of five fields per kidney from five different mice were counted. Differences between WT and E359K mice (∗) were significant (*p* < 0.01).

We analyzed kidneys from 1-year-old PBS and K220M mice and the E359K mice just prior to death (Fig. 1, *A*–*D*). The K220M kidneys were normal and indistinguishable from control mice at all time points analyzed. The PBS mutant kidneys displayed a subtle branching morphogenesis defect characterized by less tubules in the kidney papilla (Fig. 1*C*). By contrast, the E359K mice either developed obstruction or the kidneys were severely dysplastic and hypoplastic (Fig. 1*B*), consistent with defects seen in the UB-ILK-null mice (7). Abnormalities in the E359K kidneys were evident at all embryological developmental stages, and at E15.5 the kidneys were noted to be small with a moderate branching morphogenesis defect and impaired metanephric mesenchyme induction (Fig. 1, *E* and *F*). Kidneys at P0 were hypoplastic and dysplastic (Fig. 1, *G* and *H*), and they became dilated over time due to the intraluminal obstruction in the collecting ducts (Fig. 1, *I*–*L*). During the postnatal period, the tubules did not have any cell survival defects, but displayed intratubular proliferation characterized by increased Ki67 cells (Fig. 1, *M*–*O*), similar to that seen in UB-ILK-null mice (7). Thus, introducing the K220 M mutation does not cause any detectable developmental abnormalities in the collecting system, and the PBS mutation causes a subtle nonprogressive branching defect with no evidence of obstruction. On the other hand, the E359K mutation causes a severe developmental phenotype that is indistinguishable from the UB-ILK null mice (7).

### E359K, PBS, and K220M ILK mutants affect kidney CD cell tubulogenesis *in vitro*

**Figure 2:**
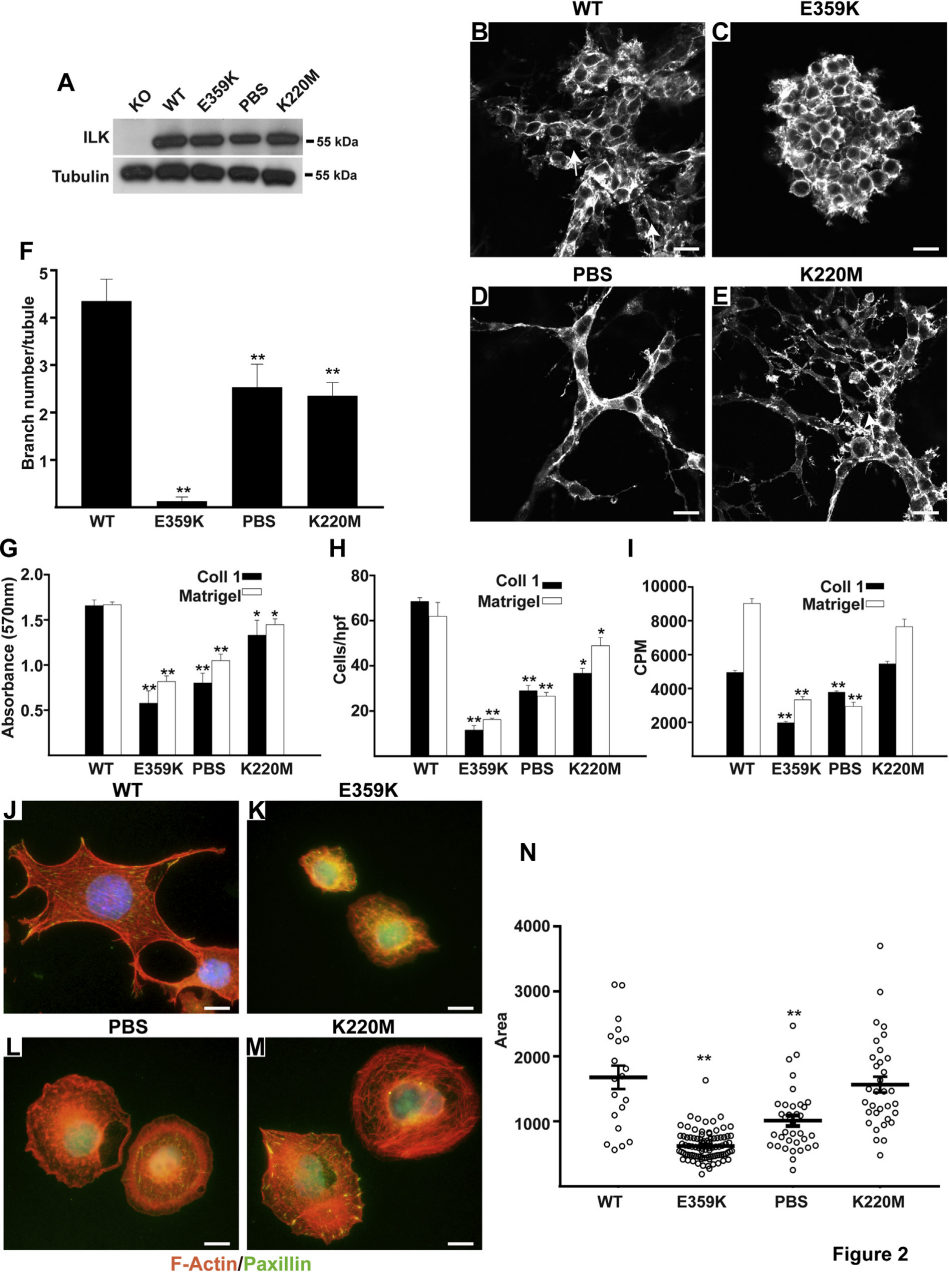
**ILK mutants differentially regulate kidney collecting duct cell functions.***A*, ILK WT and mutants were transfected into ILK-null (KO) kidney CD cells, and clones were selected for equal expression of the mutants. *B–E*, WT and mutant CD cells were placed in 3D collagen I/Matrigel gels for 7 days in the presence of 5% FBS, stained with rhodamine-phalloidin, and visualized by confocal microscopy. The *arrows* depict lumens present within the tubules. Scale bar 50 μm. *F*, the number of branches of at least 50 tubules per genotype was quantified and the average branch number ± SD is shown. Differences between WT and the E359K, PBS, and K220M mutants (∗) were significant (*p* < 0.01). *G*, CD cells were allowed to adhere to collagen I (Coll 1) or Matrigel for 1 h. Data are mean ± SD of three experiments in triplicate. ∗*p* < 0.05 (between wild type and K220M mutant and ∗∗*p* < 0.01 (between WT and E359K or PBS mutants). *H*, CD cells were plated on transwells coated with Coll 1 or Matrigel and migration, measured as cells per high-powered field (hpf), was evaluated after 4 h. Data are mean ± SD of three experiments in triplicate. (∗) and (∗∗) are as in *G*. *I*, CD cells were plated on Coll I or Matrigel. After 24 h, the cells were treated with [^3^H] thymidine and incubated for a further 24 h after which thymidine incorporation (in cpm) was determined. Data are mean ± SD of three experiments in triplicate. ∗∗*p* < 0.01 between WT and E359K or PBS mutants. *J–M*, CD cells were plated on Matrigel in serum-free medium for 4 h after which they were fixed and stained with rhodamine phalloidin and anti-paxillin antibody. Scale bar 5 μm. *N*, the area of at least 50 individual cells was measured as μm^2^. The mean cell area ± SD of the different mutant cells is shown. ∗∗*p* < 0.01 between WT and E359K or PBS mutants (N).

To define the mechanisms of action of the various ILK mutants in polarized renal epithelial cells, we generated stable clones of ILK-null CD cells (7) expressing comparable levels of wild-type (WT) ILK and the three ILK mutants (Fig. 2*A*). Multiple clones were generated and shown to have similar phenotypes. We initially performed branching morphogenesis assays in three-dimensional collagen I/Matrigel gels. CD cells expressing WT-ILK were able to form tubules with lumens (arrow) that were visible in single-plane confocal pictures (Fig. 2*B*). By contrast, the E359K CD cells were unable to undergo branching morphogenesis and made multicellular aggregates within the gel (Fig. 2, *B*, *C*, and *F*). The PBS formed unicellular cellular outgrowths with no visible lumens and had half as many branched structures per tubule as the WT-ILK cells (Fig. 2, *D* and *F*). Although the K220M mutant cells developed multicellular structures with lumens (arrow), they had half the branch number of the WT-ILK cells (Fig. 2, *E* and *F*). We next investigated well-described integrin/ILK-mediated functions, which include cell adhesion, haptotactic cell migration, cell proliferation, and spreading on extracellular matrices (Fig. 2, *G*–*N*). In all these functional assays, the E359K-CD cells had the most severe defects when plated on either collagen I or Matrigel. The PBS mutant cells showed less severe, yet significant defects, while the K220M cells showed the least severe phenotype with a mild yet significant adhesion and migration defect, but no differences in proliferation or spreading when compared with cells expressing WT-ILK (Fig. 2, *G*–*N*). Together these results demonstrate that the three mutations within the pseudokinase domain of ILK variably affect multiple integrin-dependent cell functions.

### E359K, PBS, and, K220M ILK mutations differentially regulate expression and formation of the IPP complex

**Figure 3:**
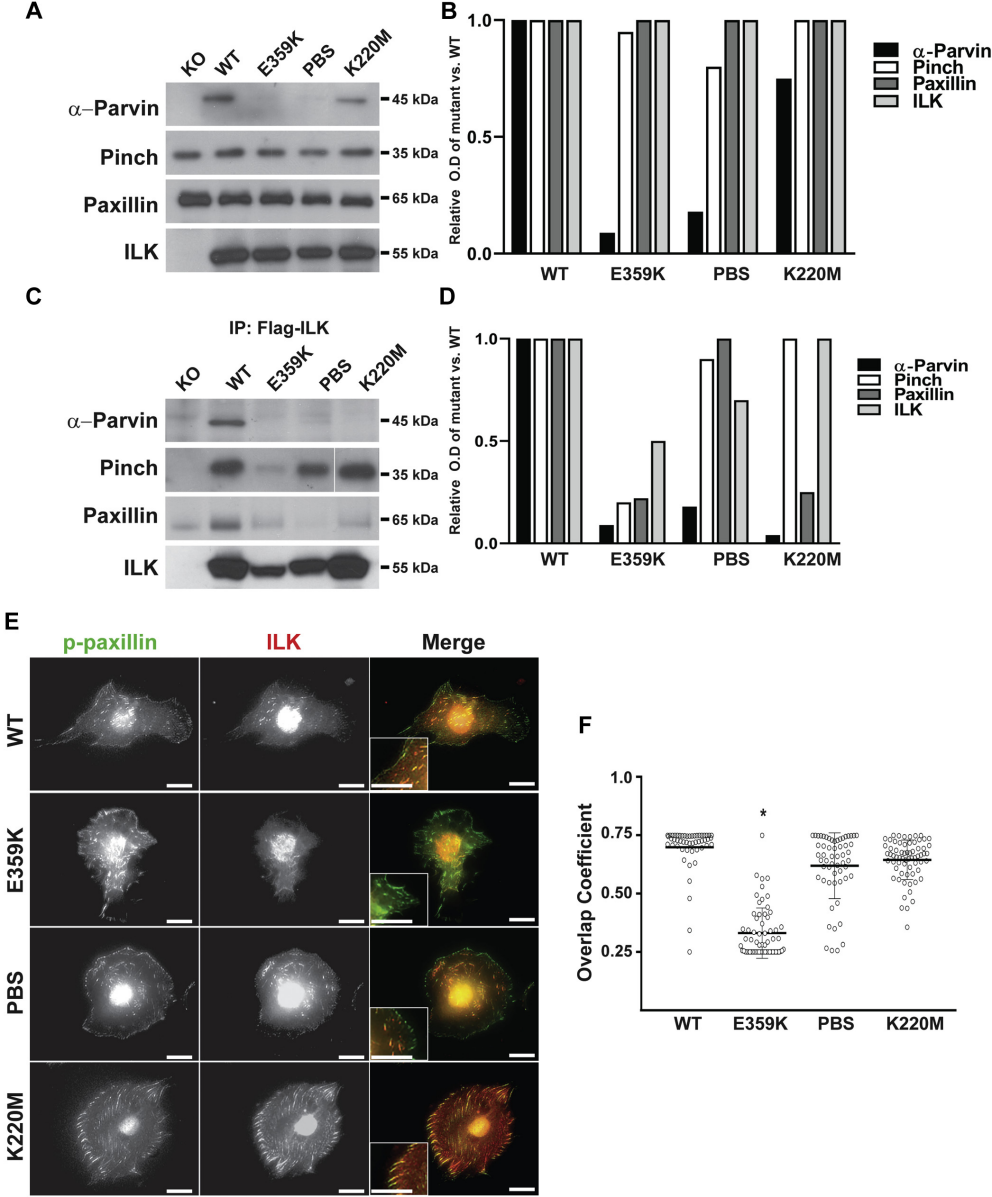
**ILK mutants differentially regulate formation of the ILK/pinch/parvin complex.***A*, immunoblots for parvin, pinch, paxillin, and ILK were performed on total cell lysates of CD cells expressing comparable amount of WT, K359K, PBS, and K220M ILK. *B*, the ratio of parvin, pinch, paxillin in the mutant cells relative to WT-ILK cells was quantified and shown graphically. A similar result was obtained in at least three independent experiments. *C*, cell lysates from the cells indicated were immunoprecipitated with anti-FLAG antibodies and the immunoprecipiated proteins blotted for parvin, pinch, paxillin, and ILK. The *solid white line* represents lane splicing from the same gel. *D*, the ratio of parvin, pinch, paxillin in the mutants relative to WT-ILK CD cells was quantified and shown graphically. *E*, CD cells expressing WT or mutant ILK were plated on Matrigel for 4 h after which they were fixed and stained with an antibody directed against phospho-paxillin (*green*) or ILK (*red*). Scale bar, 5 μm. *F*, photographs of individual cells were taken, and the amount of overlap expressed as the overlap coefficient between ILK and phospho-paxillin was determined. At least 50 cells of each genotype were counted. The mean coefficient ± SD of the different mutant cells is shown. ∗*p* < 0.01 between WT and E359K mutants.

We next investigated the mechanisms whereby the mutants induce their different phenotypes in CD cells. We initially defined the effects of the various ILK mutants on the levels of the major components of the IPP complex and paxillin, which is thought to bind to ILK *via* the highly conserved PBS site. Immunoblots of total cell lysates of CD cells expressing WT-ILK and the K220M mutation demonstrated that all three components of the IPP complex as well as paxillin were present (Fig. 3, *A* and *B*); however, similar to cells lacking ILK (KO), there was almost no α-parvin in the lysates of the E359K and PBS mutants (Fig. 3, *A* and *B*). Similar results were observed for β-parvin, which is also expressed in CD cells (Fig. S2), demonstrating that there is no compensation by β-parvin for the decreased α-parvin. There were no differences in the amount of pinch or paxillin in cells expressing WT-ILK or any of the mutants (Fig. 3, *A* and B). We next defined which proteins formed a complex with ILK by performing 12 h immunoprecipitation assays with an antibody directed against FLAG. Similar amounts of ILK were immunoprecipitated from WT- and K220M-ILK expressing cells; however, less was immunoprecipitated from the PBS and E359K mutants, respectively, suggesting there may be degradation of these mutant forms of ILK. Almost no α-parvin or β-parvin, pinch, or paxillin was immunoprecipitated with the E359K mutant (Fig. 3, *C* and *D* and Fig. S2). By contrast, pinch, but not α-parvin, β-parvin, or paxillin, was coimmunoprecipitated with the PBS or K220M mutants. We next examined whether the mutant ILK proteins localize to focal adhesion on cells that were plated on collagen I by performing costaining with ILK and phospho-paxillin antibodies. Interestingly, the WT-ILK as well as PBS and K220M mutants colocalized with phospho-paxillin (Fig. 3, *E* and *F*); however, there was almost no colocalization between the E359K mutant and phospho-paxillin, except in large paxillin focal adhesions (Fig. 3, *E* and *F*). Thus, E359K-ILK is absent from most focal adhesions and does not form a complex with parvin, paxillin, and pinch. By contrast, the PBS and K220M mutants bind with pinch and localize to focal adhesions even though they do not form a complex with paxillin or parvin.

### Mutations in the ILK pseudokinase domain result in increased turnover of the IPP complex

**Figure 4:**
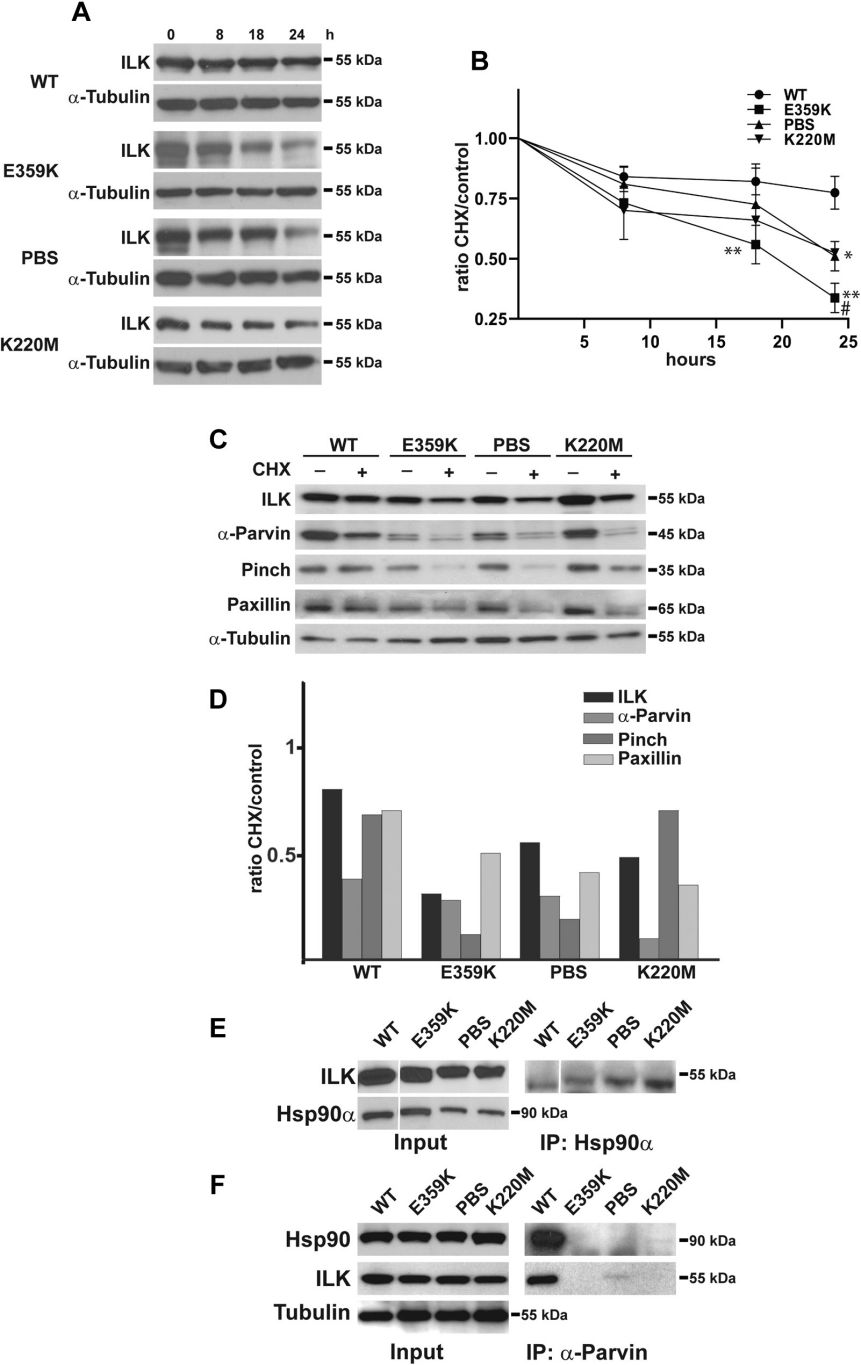
**Mutant IPP complexes turnover rapidly in an Hsp90α-independent manner.***A*, WT, E359K, PBS, and K220M ILK mutant CD cells were treated with cycloheximide and cell lysates were collected at 8, 18, and 24 h after treatment and analyzed by western blot for levels of ILK. α-Tubulin was utilized as a loading control. *B*, the ratio of signal of cycloheximide-treated cells for 8, 18, and 24 h relative to cells collected at time 0 normalized to tubulin (*y*-axis) is shown. Data are mean ± SD of three experiments in triplicate. ∗∗*p* < 0.01 between WT and E359K mutants at 18 and 24 h; ∗*p* < 0.01 between WT and PBS or K220M mutants at 24 h; #*p* < 0.01 between E359K and PBS or K220M mutants at 24 h (*C*) WT, E359K, PBS, and K220M ILK mutant CD cells were treated with vehicle or Cycloheximide. After 24 h cell lysates were analyzed by western blot for levels of ILK, α-parvin, pinch, and paxillin. α-Tubulin was utilized as a loading control. *D*, the ratio of signal of cycloheximide-treated relative to vehicle-treated cells normalized to tubulin for each mutant cell line (ratio CHX/control) is shown. This is an example of a single experiment that was repeated at least three times with similar results. *E*, the lysates of CD cells expressing WT-ILK or the mutants were immunorprecipitated with an antibody directed against HSP90α, and the immunoprecipiated proteins were analyzed by western blot for the presence of ILK. The *solid white line* represents lane splicing from the same gel. *F*, lysates of CD cells expressing WT-ILK or the mutants were immunorprecipitated with an antibody directed against α-parvin, and the immunoprecipitated proteins were analyzed for the presence of ILK. WT but not mutant ILK was pulled down by the α-parvin antibody.

We previously showed that the cellular lifetime of the PBS mutant is decreased relative to WT-ILK (12). We therefore dermined WT, E359K-, PBS-, and K220M-ILK levels in CD cells 8, 18, and 24 h after treatment with cylohexamide (to inhibit protein synthesis). There was an approximately 20% decrease of WT ILK at 8 h, which stayed relatively constant until the 24 h time point. By contrast, the amount of E359K decreased by about 30% at 8 h, 50% at 18 h, and 68% at 24 h. The decrease in the PBS and K220M mutants was between the WT and E359K mutants at 18 and 24 h (Fig. 4, *A* and *B*). The half-lives of WT, E359K-, PBS-, and K220M-ILK are 63.7, 15.5, 24.1, and 24.7 h, respectively. As the difference in amounts between all the mutants and WT ILK and between the E359K mutant and the PBS or K220M mutant was significant at 24 h, we assessed the amounts of α-parvin, pinch, and paxillin in the various cell lines at this time point. Cycloheximide decreased α-parvin by approximately 60% in the CD cells expressing WT-ILK, 75% in the E359K and PBS mutants, and 90% in the K220M mutants (Fig. 4, *C* and *D*). Interestingly, cycloheximide decreased pinch by 30% in WT and K220M ILK cells, but this was markedly decreased by 90% and 80% in the E359K and PBS mutants, respectively (Fig. 4, *C* and *D*). Finally, cycloheximide decreased paxillin by 30% in ILK WT CD cells, and it was down by 50%, 60%, and 70% in the E359K, PBS, and K220M CD cells, respectively (Fig. 4, *C* and *D*). Thus, E359K-ILK was turned over quicker than WT-ILK or the other two ILK mutants. Furthermore, α-parvin and pinch turnover was the highest in this E359K mutant followed by the PBS mutant, while in the K220M mutant α-parvin was primarily decreased.

Cellular ILK levels are regulated by the Hsp90–Hsc70 chaperone machinery where Hsp90 binds the pseudokinase domain of ILK (19). Therefore, we tested whether the increased ILK turnover in the E359K, PBS, and K220M mutants was due to a disruption between the mutated ILK proteins and Hsp90. When we immunoprecipitated Hsp90α from the ILK-WT, E359K-, PBS-, and K220M-CD cells and immunoblotted the immunoprecipitated proteins with ILK antibody, ILK was detected in all the cell populations (Fig. 4*E*). When we immunoprecipitated α-parvin in WT-ILK expressing CD cells, both ILK and Hsp90 were in the immunoprecipitated proteins; however, as expected based on the results shown in Figure 3*D*, this interaction did not occur in the mutants, where α-parvin is not part of the IPP complex (Fig. 4*F*). Thus, the mutations in ILK do not alter its ability to bind Hsp-90, which suggests that the increased turnover of the ILK mutants is not due to altered interactions between ILK and the Hsp90–Hsc70 chaperone machinery.

### Overexpression of the E359K mutant does not revert the E359K CD cell phenotype

**Figure 5:**
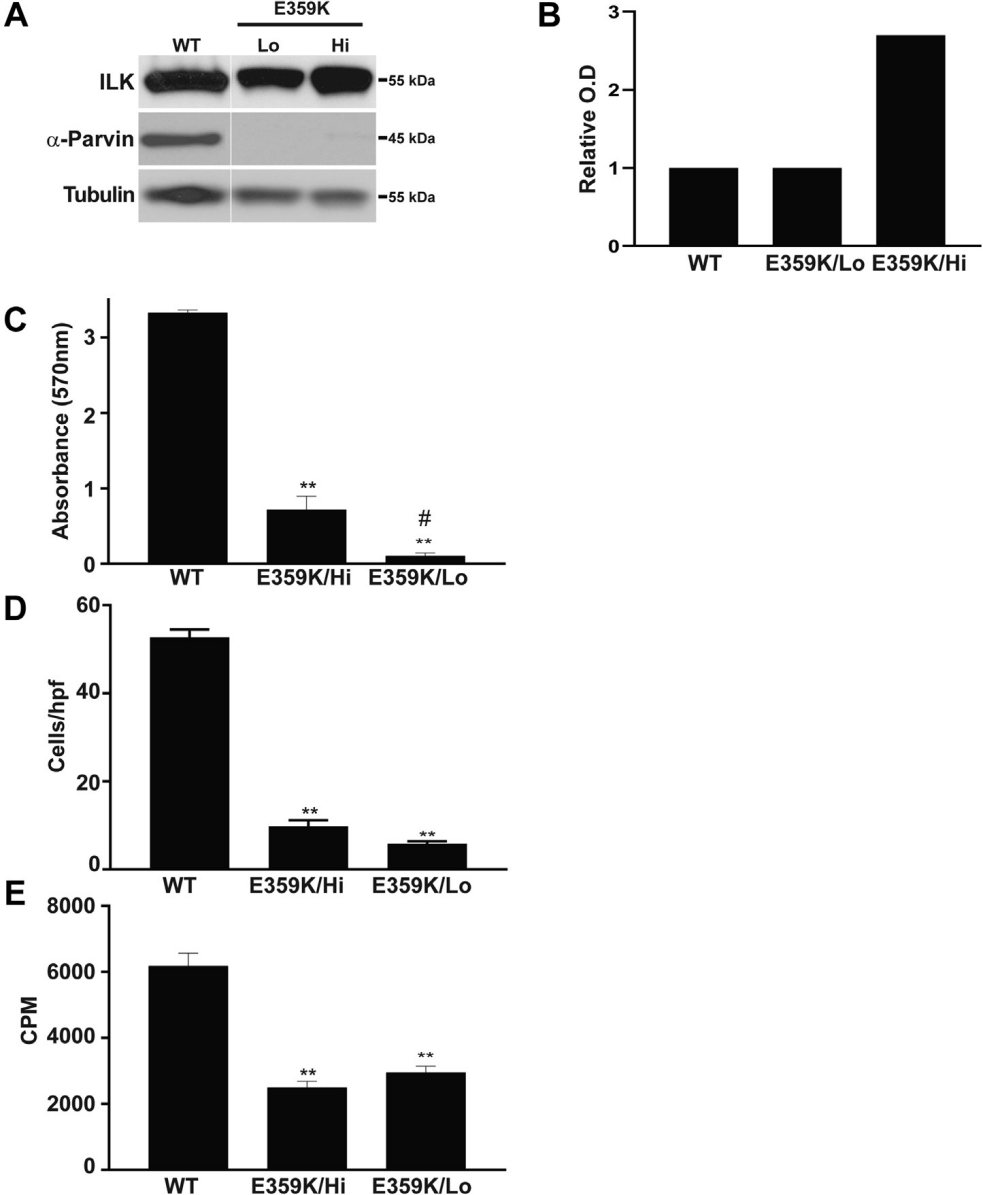
**Overexpression of the E359K mutant does not reconstitute CD cell function.***A*, CD cells with similar levels of WT and E359K-ILK (E359K/Lo) or 2.5 times the amount or E359K (E359K/Hi) were generated. The *solid white line* represents lane splicing from the same gel. *B*, the relative expression of ILK in the cells is depicted graphically. (*C*) cell adhesion, (*D*) migration, and (*E*) proliferation were performed as described in Figure 2. ∗∗*p* < 0.01 between WT and E359K and #*p* < 0.05 between E359K Hi and E359K Lo.

As E359K-ILK turnover was significantly higher than either WT-ILK, PBS, and K220M mutants, we tested whether the severe phenotype of this mutant was due to insufficient expression in the cells. We generated CD cells that expressed comparable amounts of WT-ILK and the E359K mutant (E359K/Lo) as well as CD cells that express 2.5 times more E359K-ILK (E359K/Hi) compared with WT-ILK cells (Fig. 5, *A* and *B*). In contrast to cells expressing WT-ILK, neither of the E359K mutants expressed α-parvin (Fig. 5*A*). When adhesion, migration, and proliferation assays were performed, we observed that although E359K/Hi CD cells adhered significantly more than the E359K/Lo CD cells to collagen; they still adhered significantly less than cells expressing WT-ILK (Fig. 5*C*). The E359K/Hi CD cells did not migrate or proliferate more than the E359K/Lo CD cells (Fig. 5, *D* and *E*). Thus, the functional defects observed in cells expressing E359K-ILK are likely due to disruption of global ILK structure that renders it unable to bind to either parvin or pinch.

### Mutations within the ILK pseudokinase domain induce structural perturbations

**Figure 6:**
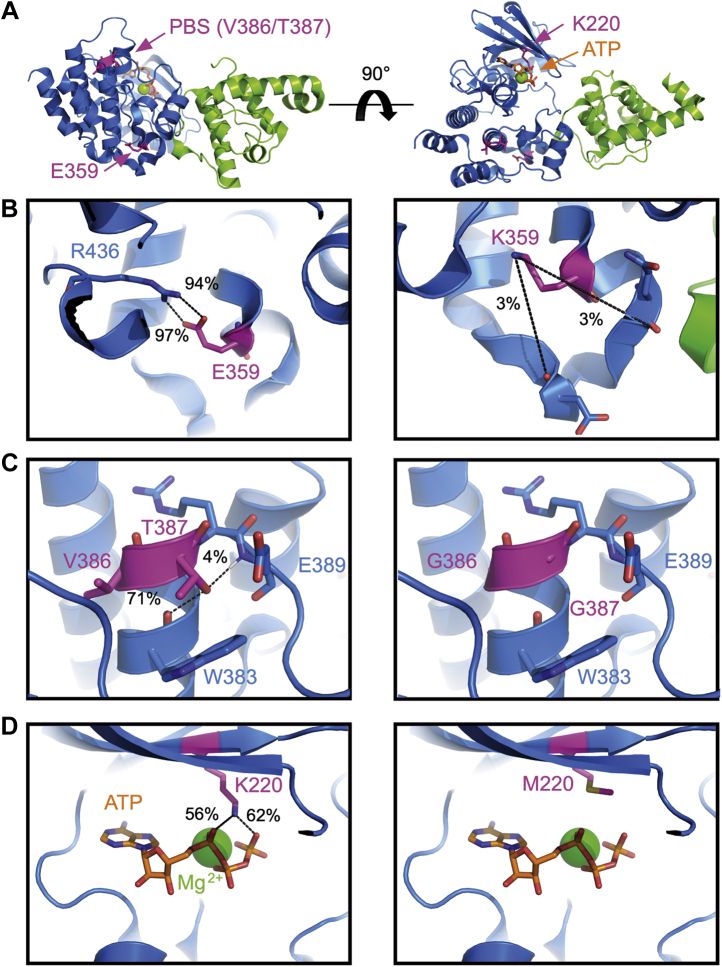
**ILK mutations disrupt intramolecular stabilization.***A*, three mutations (magenta) in ILK (blue) were analyzed by MD simulations. The sites of these mutations—E359K, PBS (V386G/T387G), and K220M—are not in direct contact with parvin (*green*). *B*, E359 forms a stable salt bridge with R436 (left panel). The E359K mutation removes this salt bridge resulting in 97% loss of hydrogen binding between the lysine side chain and varied backbone oxygens (right panel). *C*, for PBS, the combination of V386G/T387G is essential for paxillin binding, such that this double mutant is referred to as the “paxillin binding site” (PBS) mutant. The T387 OG1 side-chain atom hydrogen bonds with the E389 backbone hydrogen with 71% occupancy and with the W383 backbone oxygen with 4% (left panel). The T387G mutation is unable to facilitate these interactions (right panel). *D*, with regard to the K220M mutant, it is seen that the WT K220 NZH side-chain nitrogen makes extensive contacts with ATP (*orange*) α- and γ-phosphate group oxygen atoms. Composite occupancies with α-phosphate oxygens or γ-phosphate oxygen atoms were seen to be 56% and 62% in WT trajectories, respectively (left panel); no interactions with the β-phosphate group were observed. The K220M mutation replaces the electropositive lysine side chain with a nonpolar methionine that was not observed to interact with ATP atoms (right panel). A complete list of hydrogen-bonding occupancies is provided in Figure S2.

Our results suggest that the E359K, PBS, and K220M mutations caused increased ILK turnover, decreased the amount of parvin (E359K and PBS mutations), and resulted in an inability to form an IPP complex. To better understand the mechanism, we utilized MD simulations of each individual mutation derived from starting coordinates of the WT ILK α-parvin crystal structure (Fig. 6*A*) (3). In total, 100 ns trajectories were analyzed to determine the impact of each mutation on global structural features as well as hydrogen bonding networks relative to the WT trajectory. The E359K mutation is located in the APE motif of the putative kinase domain, which forms a conserved salt-bridge with R436 within the GHI-subdomain, which comprises the short G, H, and I helices, (Fig. 6*B*) with a hydrogen occupancy of 97% and 94% for the E359:OE1-R435:H12 and E359:OE2-R435:H22 bonds, respectively. ILK homology with other eurokeyotic protein kinases (EPKs) indicates that E359 is part of the activation segment found in canonical kinases. In addition, the GHI subdomain is an integral element of the EPK allosteric network that contains the R436 residue of the salt bridge (13). The E359K mutation results in disruption of this highly stable and well-conserved salt bridge as there is now minimal hydrogen binding (<5%) between the lysine sidechain and local backbone oxygens (20) (Fig. 6*B*). These results suggest that an E359K mutation significantly impacts the ILK molecule's structural stability that impacts multiple possible downstream effects.

Paxillin binding is reported to be disrupted in the PBS mutant (10, 11, 12). Although paxillin was not included in the simulations, the MD trajectory predicts that the T387 OG1 side-chain oxygen forms hydrogen bonds with the E389 backbone hydrogen (71% occupancy) and with the W383 backbone oxygen (<5% occupancy, Fig. 6*C*) in the WT system. The PBS mutation replaces V386 and T387 with glycines, which disrupt the hydrophobic interactions of the Val side chain and hydrogen bonds mediated by the Thr hydroxyl side chain. Of interest, a backbone hydrogen bond between the carboxyl oxygen of W383 and amide proton of G387(W383:O-G387:H) was not observed during the PBS MD trajectory. It seems unlikely that loss of this hydrogen bond would yield a global effect on ILK structure. Rather, in conjunction with less favorable hydrophobic interactions with V386G, the loss of the T387G intrahelix hydrogen bond almost certainly disrupts the local stability of the small helix comprising the paxillin binding site and likely affects the overall thermal stability of the ILK molecule.

Although ILK has no kinase activity (3), it requires ATP for its cellular function (21). In the K220M *versus* WT simulation, the K220 amide side chain was observed to participate in multiple hydrogen bonding with the α- and γ-phosphate oxygen atoms on the bound ATP molecule. Each phosphate group has multiple oxygen atoms that may interact with the K220 sidechain. The sums of the α-phosphate hydrogen bond occupancies with K220:NZH were 62% and 56% for the γ-phosphate; no interactions were observed between K220 and the β-phosphate of ATP. The K220M mutation replaces the electropositive lysine side chain with a nonpolar methionine that does not hydrogen bond with any atoms on the ATP molecule (Fig. 6*D*). A complete list of hydrogen-bonding occupancies over 10% is provided in Figure S3. Earlier studies show that K220M can alter ILK thermostability (22). Our results predict that K220M directly impairs ATP binding. Although ILK is not a kinase as defined by ATP hydrolysis, it appears that ATP binding confers a requisite level of structural stability to promote function, which if lost impacts pinch/parvin binding (21).

In sum, each mutation altered unique hydrogen bonding networks within the ILK molecule to disrupt its structure to varying degrees. More specifically, E359K broke an internal stabilizing salt bridge linked to the global structure of the protein, while PBS destabilized a small helical region known to be important in paxillin binding, and K220M (also more locally) eliminates a series of ATP-stabilizing H-bonds. Time-averaged root-mean-squared deviation (RMSD) values of each ILK subdomain are indicative of structural disruption: WT; 1.96 Å, E359K; 2.14 Å, K220M; 2.10 Å, PBS, 2.07 Å.

### Mutations within the ILK pseudokinase domain can impact contact with α-parvin

**Figure 7:**
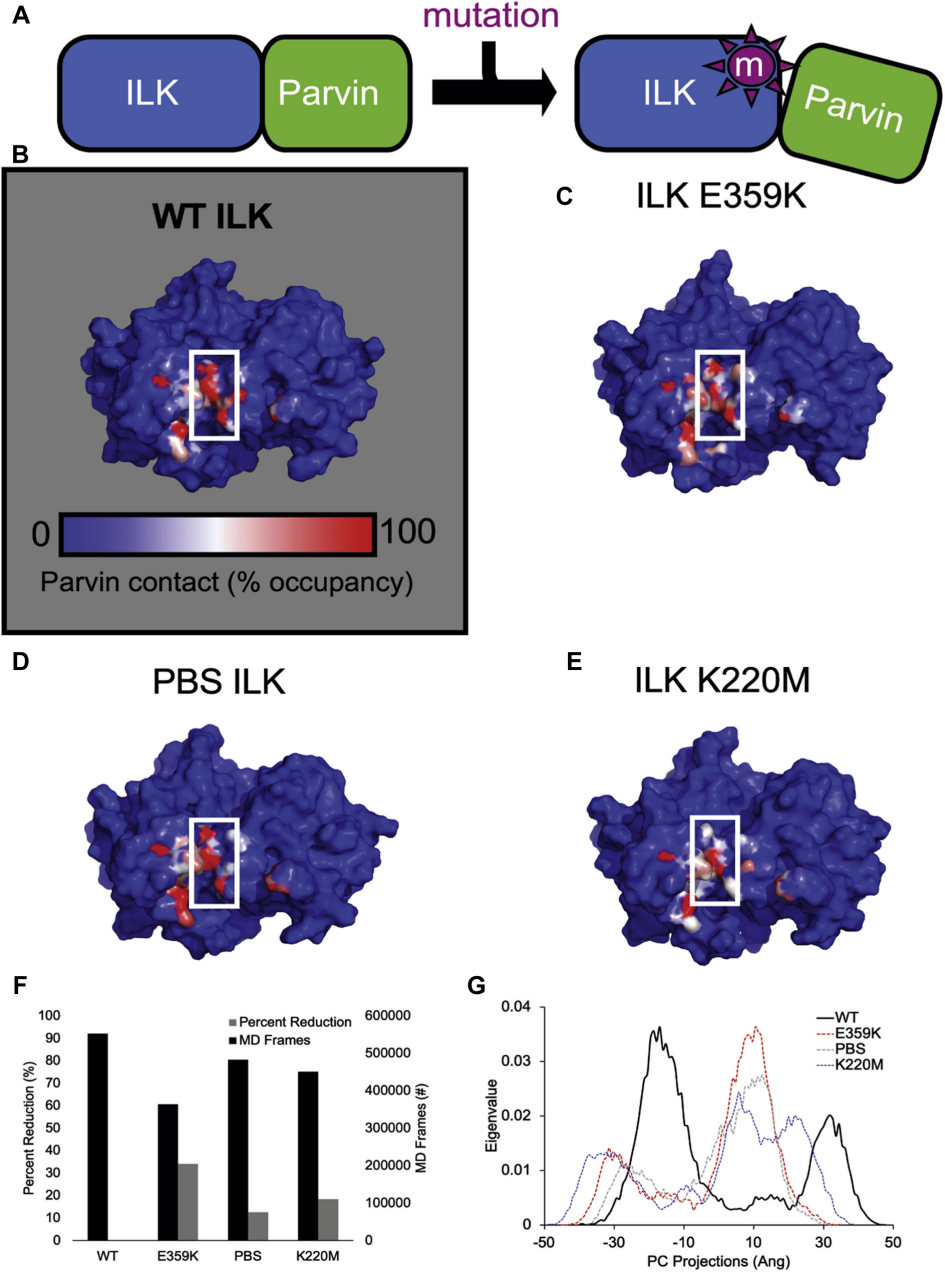
**Mutating the ILK pseudokinase domain impact contact with parvin.***A*, MD simulations predict that the mutants in this study impact the stability of the ILK-Parvin complex *via* allosteric effects, not direct contact of the mutation sites with parvin. For each mutant the percent occupancy of contact residues was calculated (1-(Mutant H-bond occupancy/WT H-bond occupancy) ∗ 100) and depicted colorimetrically on the ILK molecule (parvin not shown). A complete list of contact residues and occupancies is provided in Figure S3. Results for WT ILK (*B*) were compared with E359K (*C*), PBS (*D*), and K220M (*E*) mutant forms. A key region of ILK–parvin contact was found along an electropositive groove located down the center axis of the ILK molecule (*white box*). *F*, summation of individual atom contacts per MD frame relative to WT indicate mutations (*black bars*) produces a reduction in ILK-parvin contact (*gray bars*). *G*, a principal component analysis (PC) was employed to monitor mutation-induced changes to internal dynamics for each mutant relative to WT. The first three PCs for each mutant and WT were calculated and can be found in Figure S5. The first PC represents the largest dynamic component of the simulation. PC 1 for each MD system was depicted as normalized histograms for comparison where the eigenvalues, *i.e.*, the weight of each PC, are plotted along the ordinate (right panels).

Although molecular modeling indicates that the E359K, PBS (V386G/T387G), and K220M mutations are not found within the α-parvin-binding site (Fig. 6*A*), we theorized the mutations may induce their disruptive effects through protein allostery. To test this hypothesis, we measured hydrogen-bonding contact occupancy between ILK and α-parvin over the course of our MD trajectories (Fig. 7*A*). Percent occupancy of contact residues was calculated and depicted colorimetrically on the ILK molecule. When WT (Fig. 7*B*) was compared with E359K (Fig. 7*C*), PBS (Fig. 7*D*), and K220M (Fig. 7*E*), an important contact region comprising a slightly electropositive groove (+2 K_b_T/e_c_) located along the center axis of the ILK molecule was located (Fig. 7, white box, see also Fig. S4). This area contains the highest occupancy of α-parvin contacts where Poisson–Boltzmann analyses indicate that mutants do not affect the ILK–α-parvin electrostatic topology. A comparative summation of contact MD trajectory frames was tabulated. These results predict that the E359K mutation reduced ILK contact with α-parvin by 34%. The PBS mutation reduced contact 12.6% while the K220M mutation reduced intermolecular contact by 18.4% (Fig. 7*F*), clearly characterizing E359K as the most disruptive of the mutations studied.

Transmission of signals to distal locations on a protein that alter function is a hallmark of allostery. As a means of exploring the changes in ILK allostery introduced by E359K, PBS, and K220M mutations, a principal component (PC) analysis was performed for the first three PCs (Figs. 7*G* and S5). The coordinate covariance matrix is calculated from the time series of 3D positional coordinates and serves as input to the PC analysis (Fig. S5). Multiple studies of varied enzyme systems have demonstrated that the domain movements can be illustrated by one or a few normal modes (23, 24, 25, 26). We compare the first PC since it represents the dominant motion within each trajectory. One can see that PC 1 for WT contains two peaks, with the more dominant centered on –15 Å. The mutant PC1 is of divergent shape indicating that each single point mutation has the potential to affect the dominant modes of motion within ILK, likely driving resultant contact populations with α-parvin. Although this technique does not explicitly identify the amino acid network involved in ILK allostery, these data suggest that each individual mutation is capable of altering the internal dynamics of the ILK structure and is significantly divergent from WT (Fig. 7*G*). In addition, none of the mutants affected the electrostatic surface topology of ILK, but they are predicted to introduce discrete changes to H-bonding and Van der Waal contacts on the ILK binding surface. PC analysis supports the notion that each mutant is allosterically connected to the α-parvin-binding surface to some degree. Although we concede that protein allostery is not the only force behind parvin dissociation, it is probably a contributing factor.

## Discussion

Attempts to determine the molecular function of ILK have been confounded in the recent past by viewing data through a lens that assumes ILK is a kinase, which is now known to be false (3). In this study, we used an identical strategy to introduce ILK E359K, PBS, and K220M mutants into the developing UB and CD kidney cells that were null for ILK to define how ILK regulates cell function *via* a kinase-independent mechanism. We provide evidence that the E359K, PBS, and K220M mutants developed progressively less severe phenotypes that correlated with their rate of turnover, in rank order. Moreover, cell-based assays show different patterns of mutation-induced IPP complex disruption while MD simulations depict distinct mutation impacts on local structural features within the ILK molecule itself.

### The E359K mutation impacts the global structure of ILK to cause severe *in vivo* and *in vitro* phenotypes

The ILK E359K mutant, originally thought to abolish kinase activity *in vitro* (27), was used as a “kinase dead” ILK in overexpression studies (11, 14, 15, 28, 29, 30). A subsequent bioinformatic analysis of protein kinases implicates the structural features associated with E359 with multiple human diseases emphasizing its importance in ILK function (31). In the current study, this mutant resulted in the most significant phenotype in mice with a moderate branching morphogenesis defect followed by obstruction due to continued intraluminal cell proliferation in late development. This phenotype was identical to that observed when we deleted ILK from the UB (7) and suggested that the E359K mutant was not functional in the developing UB. This contrasts with data in zebrafish where E359K mutant ILK was able to completely reconstitute a muscle phenotype induced by deleting ILK (32). These disparities may be due to the cell type or species differences.

Our *in vitro* analysis show that the E359K mutation had the most profound functional defects, resembling those observed in ILK-null CD cells (7). In other studies, when the E359K mutation was introduced into cells expressing endogenous ILK, cell functions such as tubulogenesis (14), Akt activation (17), spreading (33), and breast cell differentiation (34) were affected. This was proposed to be due to it acting as a “kinase dead” dominant negative. Interestingly, the E359K mutant partially rescued a spreading defect in ILK-null kidney fibroblast-like cells (6), while it was completely ineffectual in our ILK-null CD cells.

The E359K mutant resulted in decreased α- and β-parvin but no change in pinch in CD cells. This result contrasts with studies on ILK-PBS-expressing fibroblasts where both pinch and α-parvin were decreased (12). It also suggests that the decrease in α- and β-parvin is due to excessive turnover rather than alterations in expression as depletion of α-parvin results in increased β-parvin expression (35). When we investigated IPP complex formation, the E359K did not interact with pinch. Further, E359K was the only mutant unable to localize to focal adhesions, suggesting that pinch is required to localize ILK to focal adhesions (9, 12). Therefore, it is likely that the E359K mutant cannot localize to focal adhesions and trigger F-actin filament bundling, which was recently shown to be mediated *via* two WASP-Homology-2 actin-binding motifs found on both pinch and α-parvin, respectively (21, 22).

Our data show that turnover of E359K relative to WT-ILK outpaced turnover of PBS and K220M in mutant cells. This was not due to loss of interactions with Hsp90α. The E359K cells also showed increased pinch and α-parvin turnover while there was no difference in paxillin turnover. These data suggest the severe functional phenotypes of the E359K-CD cells arise because this ILK mutant turns over so rapidly that the IPP complex cannot be formed. This increased turnover is proportional to the magnitude of conformational defects in the E359K ILK that disrupt its ability to form its native complexes. This is consistent with our observation that overexpressing E359K-ILK in CD cells did not increase the amount of α-parvin in CD cells and there was only a marginal improvement in cell phenotype.

Consistent with our biological studies, the E359K mutation exhibited the most disruptive effects to ILK as determined by MD. The E359 residue located within the kinase activation loop participates in a stabilizing intramolecular salt bridge with R436 (Fig. 6*B*) located within the GHI subdomain. Conservation of the E359:R436 salt bridge has been recognized within the eurekarotic protein kinase family (EPK), yet little is known about its function in ILK, a pseudokinase. Intuitively, charge reversal of Glu (E) to Lys (K) prohibits the formation of this interaction. It has been demonstrated that analogous buried salt bridges are significant contributors to protein stability (36, 37). While it is recognized that the aliphatic portions of Glu and Arg residues are significant contributors to the internal hydrophobicity of properly folded proteins (13), we do not think that is a significant contributor in this instance. Specifically, the E359 K mutant is predicted to maintain the desolvation penalty, relative to an Ala mutation, for example. However, the side-chain charge reversal targets the salt-brige linkage, pinpointing this electrostatic interaction as the source of ILK destabilization. Further, the activation loop and GHI subdomain, which are linked by the E359:R436 salt bridge, are structural features that can function as docking sites for regulatory proteins that may become dysregulated in the ILK E359 K mutant. For example, our MD simulations predict that the E359K mutation decreases overall ILK-α-parvin contact occupancy significantly relative to WT, which is consistent with the pull-down and protein turnover experiments. We would not expect complete dissociation of the ILK–α-parvin complex during our relatively short (100 ns) MD timescale, yet the early stages of dissociation may be occurring as suggested by time-averaged RMSD values of the ILK subdomain (WT 1.96 Å *versus* E359K 2.14 Å).

The activation loop and GH1 subdomins, which are teathered by the E359:R436 salt bridge, are key elements of allosteric network within the kinase family that can bind and regulate multiple substrates (20, 38, 39). Therefore, it is likely that E359K can allosterically impact parvin binding. Our PCA results are indicative of an allosteric network linking the E359K mutation and the α-parvin-binding surface of ILK. Moreover, the severity of the E359K mutation in animal and cell models in conjunction with a qualitive MD assessment suggests that E359K is allosterically linked to both pinch and parvin-binding sites (although the pinch-binding site was not included in the simulations), This may explain why, unlike PBS and K220M mutants, E359K ILK was the only mutant that failed to bind either parvin or pinch *in vivo*. An alternative explanation maybe that the E359K prohibits proper folding of ILK, in turn making it unavailable for binding pinch and parvin. However, X-ray structures of analogus mutants in cAMP-dependent protein kinase suggest that E359K may not abrogate ILK folding (13). Therefore, we posit that E359K can decrease ILK stability, thus increasing its turnover, and allosterically decrease parvin binding.

### The PBS mutant was less severe than E359K, but more detrimental than K220M

The PBS mutant causes embryonic lethality at approximately E9.5 due to defective vasculogenesis by destabilizing the pseudokinase domain (10, 11, 12). Although the PBS mutation inserted into the UB only resulted in a subtle branching abnormality, it would likely cause defective kidney development in mice constitutively expressing the mutant if they survived beyond E10.5 when kidney development occurs. Introducing the PBS mutant in CD cells resulted in an intermediate phenotype between E359K and K220M. It also resulted in decreased levels of and binding to parvin and paxillin in CD cells, while pinch levels and binding remained unaffected. This result contrasts with studies on ILK-PBS-expressing fibroblasts where both pinch and α-parvin were decreased and no IPP complex formed (12). The reason for these differences is unclear and highlights cell-type-specific functions of ILK.

We show excessive turnover of PBS relative to WT-ILK. The PBS cells also showed increased pinch and α-parvin turnover while there was no difference in paxillin turnover. Our results agree with a previous study showing decreased stability of the PBS mutant and that paxillin was not required for IPP complex formation (12). Further, PBS formed a complex with Hsp90α, suggesting that the degradation of the PBS mutants is not due to loss of interaction with this heat shock protein. Molecular modeling in combination with MD simulations predicts that the PBS mutant disrupts a stabilizing hydrogen bond and hydrophobic networks making the paxillin-binding loop more flexible and possibly decreasing overall ILK thermostabiliy. Due to the absence of paxillin in our simulation, we cannot comment on direct participation of Val or Thr in paxillin binding. However, our simulations suggest that the PBS mutation induces only a modest structural change to ILK, relative to E359K, *i.e.*, WT RMSD 1.96 Å *versus* E359K 2.14 Å, *versus* PBS, 2.07 Å. This suggests a reduction of ILK contacts with α-parvin.

### The ILK K220M mutant produces the mildest phenotype

Constitutive expression of the K220M mutation caused renal agenesis in mice because it disrupts ILK–α-parvin interactions (9). In addition, the same mutant was unable to reconstitute a muscle phenotype in ILK-null zebrafish, suggesting that this mutation rendered ILK inactive in this situation (32). The kidney phentotype was caused by inhibiting outgrowth and branching of the UB and induction of the metanephric mesenchyme due to the constitutive presence of the homozygous K220M mutation in all embryonic structures at the intitiaiton of kidney development. By contrast, in this study we show that the presence of only the K220M mutant allele in the UB, achieved by deleting the floxed WT-ILK allele with hoxb7cre, did not result in a developmental phenotype. Although the floxed ILK allele was efficiently deleted in this mouse, the ILK-mRNA and ILK-protein remained at sufficiently high levels and long enough to overcome the impact of the K220M mutation to allow for the UB-mesenchyme interaction and the induction of kidney development. Indeed, tissue recombination experiments (9) revealed that kidney development fails when the UB derived from homozygous K220M embryos was recombined with kidney mesenchyme from WT embryos, whereas kidney development proceeded without apparent defects when the mesenchyme was derived from homozygous K220M and the UB from WT-ILK embryos. Hence, it is very likely that the late loss of the WT-ILK in our current model is responsible for the mild kidney defects. However, we cannot completely exclude that the K220M mutation does not cause mild phenotypes in the UB. This possibility is supported by the mild *in vitro* tubulogenesis phenotype (Fig. 2*E*). Despite the big discrepancy in the kidney phenotype in the different mice, the cell function and biochemical phenotypes in our CD cells and the UB cells derived from constitutive K220M were similar (9, 12).

It was demonstrated that ATP binding is essential for IPP function (21). Here we show how the K220M could directly impact ATP-binding stability. Specifically, the K220 side chain stabilizes ATP binding by interacting with the oxygen atoms of the α- and γ-phosphates. Mutation of the electropositive lysine to a nonpolar methionine eliminates these ATP-stabilizing hydrogen bonds, logically reducing the binding stability of ATP, an essential cofactor for IPP function *in vivo*. This indicates that while not catalytic, ATP binding confers a structural morphology or augmented stability to ILK necessary for proper function. Further, our calculations predict that the K220M mutation can allosterically reduce ILK–α-parvin contacts. If K220M disrupts both α-parvin and ATP binding, this would in turn prohibit the requisite association of actin stress fibers to α-parvin in the focal adhesion. At this point, it seems equally likely that loss of ATP binding impacts the stability of α-parvin binding, increasing the turnover of both ILK and α-parvin.

In conclusion, we provide evidence that the K220M, PBS, and E359K mutants are associated with progressively more severe phenotypes that correlated with their degree of structural perturbations and rates of turnover; with the E359K mutant being the most highly perturbed and K220M the least. These defects correlate with the severity of IPP stability and complex formation. Our MD simulations and molecular modeling provided a measure of molecular insight predicting how each of these mutations disrupt specific intra-ILK hydrogen bond networks that uniquely disrupt the ILK-α-parvin subdomains of the IPP. We also show that while all three mutations disrupted ILK binding to parvins, only the E359K mutant also disrupted ILK binding to pinch. The simplest explanation is that the E359K mutation severely destabilizes ILK–pinch and ILK–parvin interactions, resulting in a significantly worse phenotype than the PBS and K220M mutants, which only alter ILK–parvin binding by inducing structural perturbations in ILK that are more localized than for the E359K mutant.

## Experimental procedures

### Mouse strains and morphological analysis

Mice carrying the V386G/T387G substitution and the K220M mutation were generated as described previously for other ILK mutations (9, 12). To make the E359K mutant, the GAA in exon11, which encoded E359, was mutated to AAA using site-directed mutagenesis. A loxP-flanked neomycin cassette was inserted into exon 13 of the murine ILK gene (6), and the construct was electroporated into R1 ES cells (40). Mutant ES cells were injected into C57B6 blastocysts to generate germ-line chimaeras. Mutant offspring were intercrossed with deleter-Cre transgenic mice to remove the neomycin cassette. The transgenic mice that had the point mutations in ILK were intercrossed with floxed ILK mice and hoxB7 promoter-driven Cre recombinase transgene mice (18). Mice were backcrossed at least seven times to the C57BL/6 genetic background prior to analysis. Mouse kidneys were fixed, embedded in paraffin, sectioned, stained with hematoxylin and eosin, and examined by light microscopy. KI67 staining was performed as previously described (41). Experiments were approved by the Vanderbilt University Institutional Animal Use and Care Committee.

### Generation of ILK mutant CD cell lines

ILK-null CD cells described previously (7) were transfected with either full-length, E359K, V386G/T387G, or K220M mutated FLAG-tagged ILK constructs. Clones of the transfected cells were generated and tested for equal expression of ILK. At least three clones of the different cell lines were tested, and similar results were recorded for each clone.

### Cell adhesion

Ninety-six-well plates were coated with different concentrations of collagen I or Matrigel and blocked with BSA. In total, 10^5^ cells in serum-free Dulbecco's Minimal Essential Media (DMEM) were placed in each well for 60 min; nonadherent cells were removed, and the remaining cells were fixed, stained with crystal violet, and solubilized. The optical density of the cell lysates was read at 540 nm.

### Cell migration

Transwells with 8 μm pores were coated with collagen I or Matrigel and 10^5^ cells were added to the upper well in serum-free medium. Cells that migrated through the filter after 4 h were counted. Three random fields were analyzed per each treatment.

### Cell proliferation

In total, 5 × 10^3^ cells were plated per well in 96-well plates on collagen I or Matrigel and maintained in DMEM (10% FBS). After 12 h, the cells were incubated in DMEM (2% FBS) for 24 h and then pulsed with 1 μCi/well [3H] thymidine (PerkinElmer Life Sciences). Twenty-four hours later, the cells were solubilized, and radioactivity was measured using a scintillation counter.

### Cell spreading

Cells were plated onto slides coated with collagen I (10 μg/ml) for 1 h, after which they were fixed, permeabilized, and stained with rhodamine phalloidin (1:5000) and an antibody directed against paxillin (Phospho-Paxillin; Cell Signaling cat#2541). The cells were visualized under a fluorescent microscope and the area of at least 30 individual cells was calculated using Image J software.

### Tubulogenesis assays

In total, 1.5 × 10^3^ CD cells were placed in 3D gels comprising rat tail collagen I and Matrigel (Becton Dickinson) and DMEM containing 20 mM HEPES (pH 7.2) as previously described (42). Hundred microliters of gel was plated onto 96 wells. After 1 h at 37 °C, an equal volume of DMEM supplemented with 10% FBS was added to the gels. The cells grew for 7 days, at which time the gels were stained with Rhodamine Phalloidin and photographed on a confocal microscope.

### Western blotting and immunoprecipitations

Cells were homogenized in 150 mM NaCl, 50 mM Tris-HCl, 5 mM EDTA, 0.1% w/v SDS, 1.0% (w/v) sodium deoxycholate, 1.0% (v/v) Triton X-100, phosphatase inhibitor cocktails P1 and P2, pH 7.6 (all by Sigma) supplemented with complete protease inhibitors (Roche Applied Science), followed by sonication for 30 s at 4 °C. Equal amounts of total protein per lane were separated on a polyacrylamide gel and transferred to PVDF membranes (Millipore). Membrane blocking and antibody dilution were performed with TBS, pH 7.6, supplemented with 0.1% Tween 20 (Serva) and 2% skim milk (Fluka) or 5% BSA (PAA Laboratories). Subsequently, membranes were incubated for 1 h at room temperature or overnight at 4 °C with primary antibodies to ILK (Cell signaling; cat#3856S; conjugated to either agarose or sepharose beads), phospho-paxillin (Cell Signaling; cat#2541), FLAG (M2 Sigma), α-parvin (Cell Signaling; cat#8190), β-parvin (Proteintech), HSP90 (Cell Signaling; cat# 4874), HSP90Beta (Cell Signaling; cat#5087). After washing, appropriate HRP-coupled secondary antibodies (Bio-Rad) were applied for 1 h at room temperature. After final washing, ECL detection (Immobilon, Millipore) was performed at a LAS4000 (Fujifilm). Images were analyzed with ImageJ software for quantification.

For immunoprecipitation of FLAG-tagged ILK, cells were starved overnight and then plated for 4 h on FN-coated dishes. After lysis in 10 mM Tris-HCl, 50 mM NaCl, 10% (v/v) glycerol, 1%(v/v) Igepal C630, and inhibitors as above, pH 7.6, on ice, cells were sonicated, debris was pelleted, and 1.0 mg of total protein was immunoprecipitated using M2-anti-FLAG resin (Sigma). After washing and elution, coimmunoprecipitated protein was visualized by western blotting.

### Statistics

All experiments were repeated at least three times. All numerical data are given as mean value with SD or S.E. For S.E., the number of biological replicates used is indicated in the figure legend. Statistical significance was tested by two-tailed Student's *t*-test, Chi-square, or one-way ANOVA tests.

### Molecular dynamics (MD) simulations and molecular modeling

MD simulations utilized the AMBER 16 (43) program suite and the ff14SB parameter set (44). Starting coordinates were taken from the X-ray structure of the ILK/α-parvin core complex (PDB 3KMW) (3). E359K, K220M, R211A, S343A, PBS (V386G, T387G) point mutations were introduced to the 3KMW coordinate system *via* PyMOL (The PyMOL Molecular Graphics System, Version 2.3.2). Each system was charge neutralized with the addition of sodium counter ions along a 1.0 Å Coulombic potential grid. Monovalent ion parameter sets were used as described earlier (45). MD systems were solvated in a truncated octahedral box using the TIP3P water model to a distance of 8.0 Å. Energy minimization and solvent equilibration were achieved under periodic boundary conditions. Production calculations were conducted at constant pressure for 100 ns. Temperature was maintained at 300 K by a Langevin coupling algorithm using a collision frequency of 0.5 ps^−1^ (46, 47). Electrostatic interactions were treated with the particle mesh Ewald (PME) method (48). The SHAKE algorithm was used to constrain bond lengths involving hydrogen atoms. A nonbonded cutoff of 9.0 Å was set during all minimization, equilibration, and production stages. A PC analysis was conducted as described in detail elsewhere (49, 50). In brief, we calculated the coordinate covariance matrix, corrected for rotational and translations motion. The matrix was diagonlized to obtain the first three eigenvectors (PCs) that were normalized into histograms for visualization. Resultant trajectories were analyzed, and PCs were generated using the CPPTRAJ module of AMBER16. Molecular modeling and trajectory visualization were performed with PyMOL.

## Data availability

All data used to derive conclusions and facilitate discussion are contained herein.

## Conflict of interest

The authors declare that they have no conflicts of interest with the contents of this article.
