# Supplementary material for: Disruption of the integrin-linked kinase (ILK) pseudokinase domain affects kidney development in mice
Source: J Biol Chem. 2021 Feb 2;296:100361. doi: 10.1016/j.jbc.2021.100361 (PMC7949147; doi:10.1016/j.jbc.2021.100361)
Supplement: Figures S1 to S5 [file mmc1.pdf]

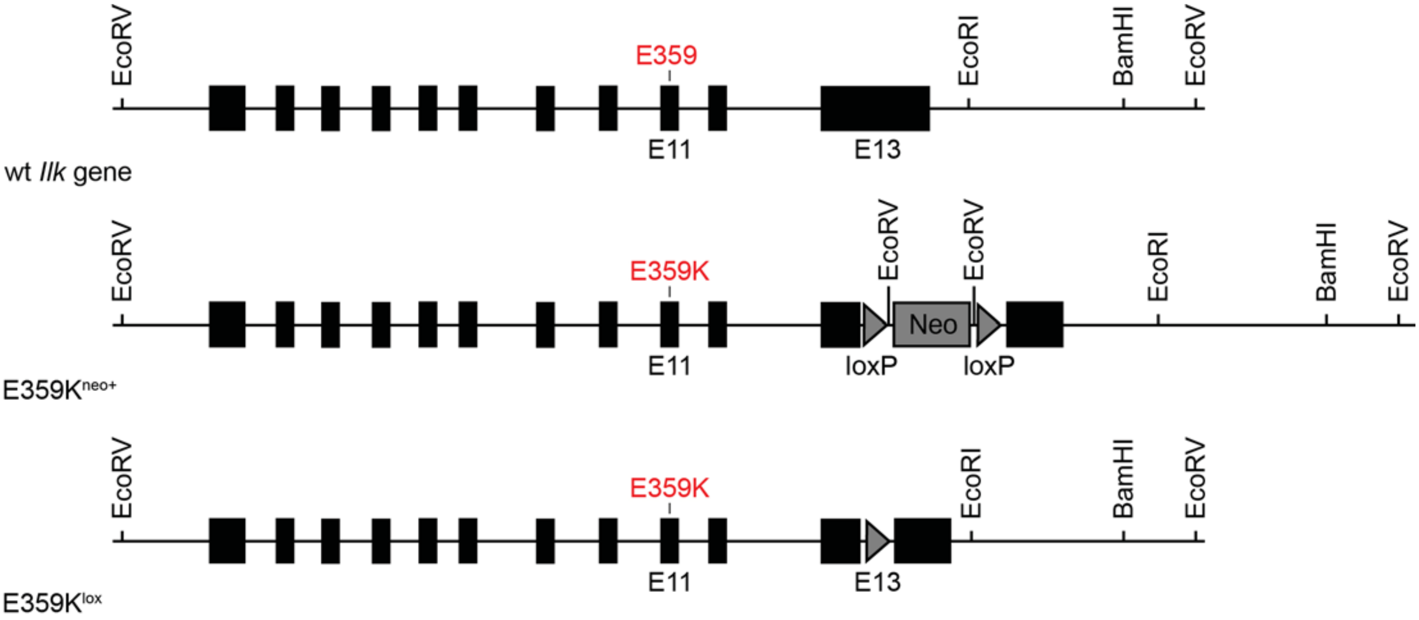

Supplemental figure 1

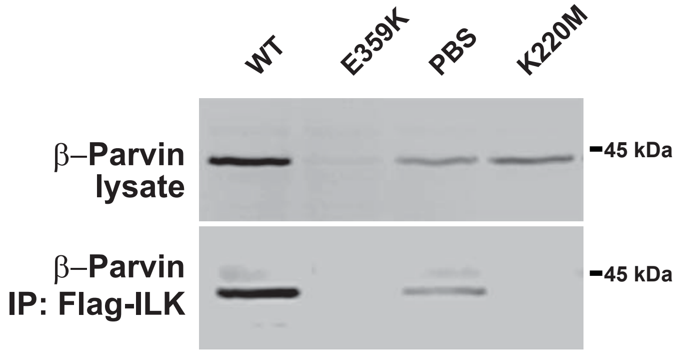

Supplemental figure 2

A

WT

Acceptor Residue	Acceptor Atom	Donor Residue	Donor Atom	Frames	Occupancy
GLU 359	OE2	ARG 436	HH12	24469	0.9788
GLU 359	OE1	ARG 436	HH22	23417	0.9367
GLU 359	OE1	GLU 359	H	21695	0.8678
GLU 359	O	LYS 363	H	19107	0.7643
GLU 359	O	GLN 362	H	8151	0.326
GLU 359	OE2	WAT	SolventH	24287	0.9715
GLU 359	OE1	WAT	SolventH	23312	0.9325

E395K

Acceptor Residue	Acceptor Atom	Donor Residue	Donor Atom	Frames	Occupancy
LYS 359	O	LYS 363	N	16131	0.6452
LYS 359	O	GLN 362	N	8517	0.3407
WAT	H	LYS 359	NZ	14433	0.5773
WAT	H	LYS 359	NZ	14336	0.5734
WAT	H	LYS 359	NZ	14247	0.5699
WAT	H	LYS 359	N	8932	0.3573

B

	Acceptor Residue	Acceptor Atom	Donor Residue	Donor Atom	Frames	Occupancy
**WT**	TRP 383	O	THR 377	HG1	17773	0.7109
	TRP 383	O	THR 377	H	13849	0.554
	THR 377	OG1	WAT	H	3080	0.1232
**PBS**	TRP 383	O	GLY 387	H	11638	0.4655
	TRP 383	O	GLY 386	H	351	0.014
	GLY 386	O	WAT	H	26081	1.0432
	GLY 387	O	WAT	H	29677	1.1871

C

WT

Acceptor Residue	Acceptor Atom	Donor Residue	Donor Atom	Frames	Occupancy
ATP 502	O1A	LYS 220	HZ3	5339	0.2136
ATP 502	O3G	LYS 220	HZ2	5334	0.2134
ATP 502	O3G	LYS 220	HZ1	5170	0.2068
ATP 502	O3G	LYS 220	HZ3	5031	0.2012
ATP 502	O1A	LYS 220	HZ1	4281	0.1712
ATP 502	O1A	LYS 220	HZ2	4205	0.1682
WAT	O	LYS 36	HZ1	2738	0.1095
WAT	O	LYS 36	HZ3	2727	0.1091
WAT	O	LYS 36	HZ2	2614	0.1046

K220M

ILK		Parvin		WT		E359K		PBS		K220M	
Residue	Atom	Residue	Atom	MD Frames	Fractional Occupancy	MD Frames	Fractional Occupancy	MD Frames	Fractional Occupancy	MD Frames	Fractional Occupancy
350	H	307	O	25000	1	24998	1	24998	1	24998	1
350	O	307	H	25000	1	25000	1	25000	1	24999	1
361	O	310	H	24740	0.99	24850	0.994	24862	0.994	24283	0.971
397	O	307	HB2	24619	0.985	24583	0.983	24647	0.986	24516	0.981
397	HD22	305	O	24303	0.972	24258	0.97	24625	0.985	22269	0.891
398	HB2	306	O	21558	0.862	20720	0.829	21313	0.853	21338	0.854
225	HH21	336	OD2	21408	0.856	19328	0.773	21506	0.86	20325	0.813
348	O	309	HD2	20655	0.826	19969	0.799	19575	0.783		
350	O	307	N	18981	0.759	19070	0.763	18534	0.741	19151	0.766
349	HA	307	O	18935	0.757			11048	0.442		
348	O	308	HA	18737	0.749	15815	0.633	18128	0.725	11631	0.465
350	N	307	O	18597	0.744					20994	0.84
350	O	306	HA	18522	0.741					16985	0.679
397	ND2	305	O	18482	0.739					18086	0.723
362	O	311	HB3	18188	0.728					16256	0.65
398	SD	307	HD1	16985	0.679			17220	0.689		
363	O	311	HD2	16744	0.67						
225	HH21	336	OD1	15542	0.622						
397	HB2	305	O	13119	0.525			13849	0.554	12049	0.482
225	NH2	336	OD2	12867	0.515			11273	0.451		
225	HH22	332	OE2	12541	0.502						
350	SD	307	HD2	11986	0.479	10623	0.425				
225	HH12	332	OE2	11419	0.457						
225	NH2	332	OE2	11264	0.451						
361	O	310	HG	10915	0.437			11707	0.468		
351	OH	306	HD2	10488	0.42						
362	O	311	HD2	9728	0.389	8027	0.321	8870	0.355	4568	0.183
350	HB3	307	O	7785	0.311	5449	0.218			3564	0.143
398	HE2	306	O	7759	0.31						
225	HE	336	OD1	7738	0.31						
361	O	307	HZ	6057	0.242						
398	HG2	298	O	5995	0.24						
399	OE1	278	HB3	5669	0.227	4594	0.184	4870	0.195		
348	O	308	HG12	5681	0.227						
397	HB3	307	N	5150	0.206						
225	NE	336	OD1	4827	0.193						
402	SD	307	HE1	4448	0.178			4676	0.187		
399	OE2	278	HD3	3488	0.14						
403	NZ	278	O	3262	0.13						
348	O	308	HG22	3164	0.127			1697	0.0679	2221	0.0888
403	HZ2	278	O	2766	0.111						
399	OE1	278	HD3	2650	0.106	872	0.0349				
225	NH1	335	HB2	2615	0.105						
225	HE	336	N	2581	0.103						
225	HH22	332	OE1			14476	0.579	12615	0.505	12640	0.506
225	HH12	332	OE1			12788	0.512	12344	0.494	11652	0.466
225	NH2	332	OE1			12471	0.499	11131	0.445	10884	0.435
350	O	307	HB3			9841	0.394				
364	HA	311	NE2			9683	0.387				
361	O	309	HB3			8050	0.322			9836	0.393
398	HE3	306	O			7559	0.302	7374	0.295		
225	NH1	332	OE1			7179	0.287	7815	0.313	6567	0.263
225	NH1	335	HB3			6487	0.259				
349	NH2	336	HB3			5978	0.239			1076	0.043
225	NE	335	HB3			5370	0.215				
365	HD3	311	NE2			4450	0.178				
363	N	311	HD2			3892	0.156				
348	O	308	HG23			3828	0.153				
403	HZ1	278	O			3516	0.141				

361	O	309	HA					20604	0.824	18607	0.744
225	HE	336	OD2					17260	0.69		
350	N	306	HB2					15767	0.631	3836	0.153
348	O	309	HD3					15293	0.612		
225	HH21	332	O					12384	0.495	12246	0.49
361	O	310	N					8772	0.351		
225	NH2	332	HB3					7648	0.306	5029	0.201
362	O	311	H					6334	0.253		
403	HE3	278	O					4764	0.191	4396	0.176
364	HA	311	ND1					3755	0.15	1782	0.0713
398	SD	298	HD23					2985	0.119		
225	HH11	335	OE1					2900	0.116		
362	HA	310	N					2699	0.108		
348	O	308	HG11					2505	0.1	7097	0.284
398	O	307	HD1							8881	0.355
225	NH2	336	OD1							8105	0.324
398	HE1	306	O							7282	0.291
398	SD	307	HA							6952	0.278
398	SD	308	H							4702	0.188
350	SD	309	HG3							4616	0.185
349	NH2	336	OD2							4129	0.165
399	OE2	278	HB3							3764	0.151
399	HG3	278	O							3627	0.145
398	HE3	308	N							2527	0.101
398	HE1	308	N							2510	0.1
		**SUM**		552958		363724		483347		450976	
		**Percent loss occupancy**				0.34222129		0.125888404		0.184429921	

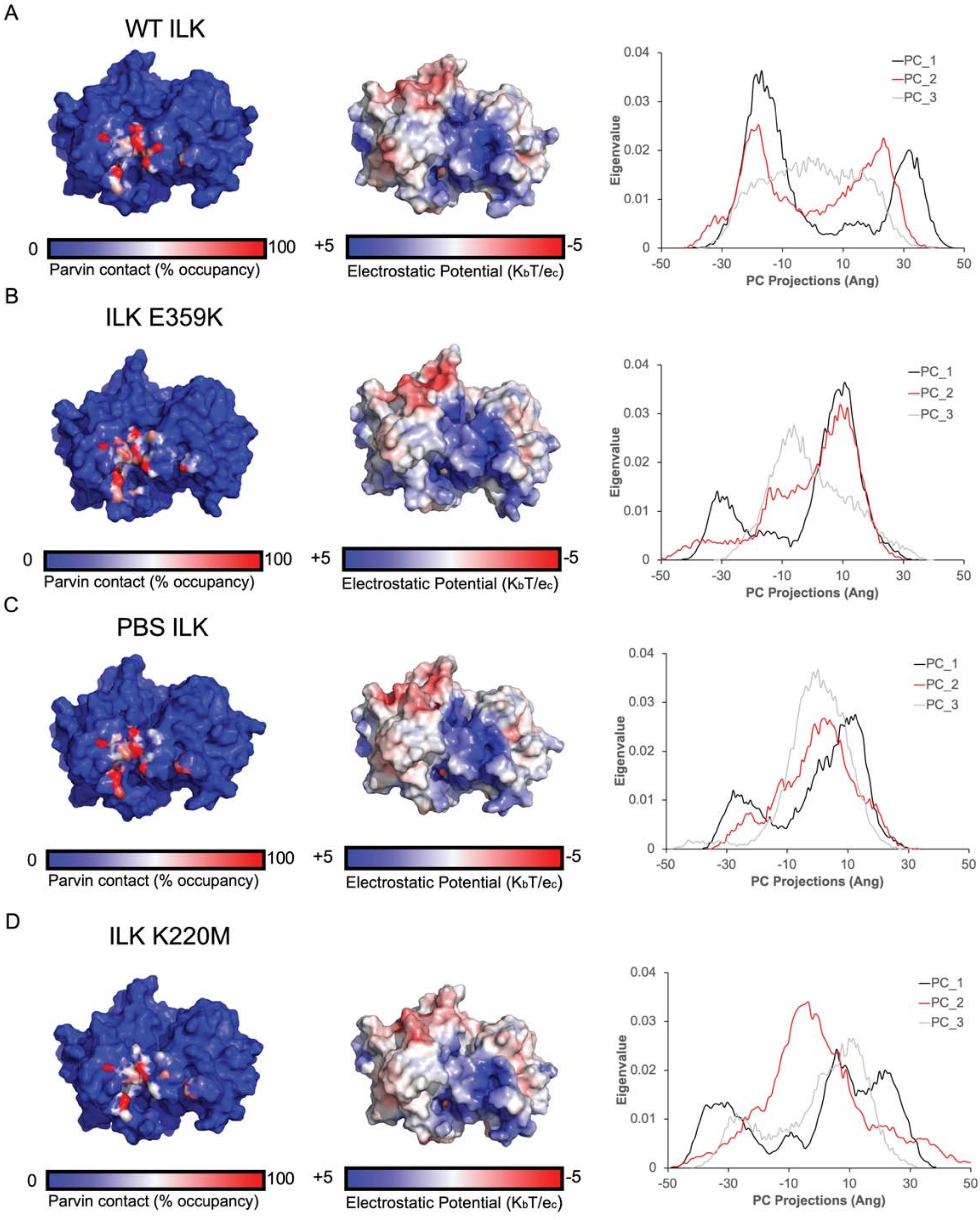

Supplemental figure 5

Supporting Information

Figure S1: Generation of ILK E359K mutant mouse. Scheme of the murine ILK locus with black rectangles indicating exons. The indicated point mutation in exon 11 was generated in a PAC (P1 artificial chromosome)-derived fragment of the ILK locus by site-directed mutagenesis. A neomycin selection cassette (neo) flanked by loxP sites (triangles) was introduced into the 3' untranslated region of exon 13 to select for positive homologous recombinant R1 embryonic stem (ES) cell clones. Correct homologous recombination was tested by Southern blotting of EcoRV-digested genomic DNA with an external probe, which binds to a 9 kB fragment in wild-type (ILK+) and 5 kB fragment in correctly targeted neo-positive knock-in (ILKki, neo) alleles. Cre recombinase-mediated excision of the neo-cassette results in a neo-negative knock-in E359K allele.

Figure S2: ILK mutants differentially express β-parvin. Top panel. Immunoblots for β-parvin, were performed on total cell lysates of CD cells expressing comparable amount of WT, K359K, PBS, and K220M ILK. Bottom panel. Cell lysates from the cells indicated were immunoprecipitated with anti-FLAG antibodies and blotted for β-parvin.

Figure S3: MD simulation ILK mutation hydrogen bond list. Native (unshaded) and solvent (grey shaded) hydrogen bonds to specific mutant residues were compare to wild type for E359K (A), PBS (B), and K220M (C).

Figure S4: ILK-Parvin contacts. ILK-parvin atomic contacts were recorded per MD frame during the 100 ns trajectory. The hydrogen bond occupancy was calculated for individual mutations relative to WT (bottom).

Figure S5: ILK electrostatic and contact surface comparison. Parvin contact maps (left panels) were generated and compared to electrostatic surfaces (center panels) and principle component histograms of the first 3 PCs (right panels) for WT (A), E359K (B), PBS (C), and K220M (D) mutations. A principle component analysis (PC) was employed to monitor mutation induced changes to internal dynamics. The first three PCs were depicted as normalized histograms, where the eigenvalues—i.e. the weight of each PC—are plotted along the ordinate.
